# Keratin 8 Is Required for the Maintenance of Architectural Structure in Thymus Epithelium

**DOI:** 10.1371/journal.pone.0075101

**Published:** 2013-09-24

**Authors:** Chikako Odaka, Anne Loranger, Kazuya Takizawa, Michel Ouellet, Michel J. Tremblay, Shigeo Murata, Akihito Inoko, Masaki Inagaki, Normand Marceau

**Affiliations:** 1 Department of Safety Research on Blood and Biological Products, National Institute of Infectious Diseases, Tokyo, Japan; 2 Centre de recherche sur le cancer de l’Université Laval, and Axe Oncologie, Centre de recherche du CHU de Québec, Québec, Canada; 3 Centre de recherche en infectiologie de l’Université Laval, and Axe Maladies infectieuses et immunitaires, Centre de recherche du CHU de Québec, Québec, Canada; 4 Laboratory of Protein Metabolism, Graduate School of Pharmaceutical Sciences, University of Tokyo, Tokyo, Japan; 5 Division of Biochemistry, Aichi Cancer Center Research Institute, Nagoya, Japan; National Cancer Institute, United States of America

## Abstract

Keratins (Ks), the intermediate filament (IF) proteins of epithelia, are coordinately expressed as pairs in a cell-lineage and differentiation manner. Cortical thymic epithelial cells (cTECs) predominantly express the simple epithelium keratin 8/18 (K8/K18) pair, whereas medullary thymic epithelial cells (mTECs) express the stratified epithelium K5/K14 pair, with TECs exhibiting K5 and K8 at the cortico-medullary junction in mature thymus. In the work reported here, we used wild-type (WT) and K8-knockout (K8-null) mice to address the contribution of K8/K18 IFs in the maintenance of the thymic epithelial structure. K8-null thymus maintained the differential cell segregation at the cortex versus the medulla observed in WT thymus, and the distribution of immature thymocytes at the cortex. The K8/K18 loss did not affect thymocyte development. However, it massively perturbed the TEC morphology both at the cortex and the medulla, along with a prominent depletion of cTECs. Such tissue alterations coincided with an increase in apoptosis and a reduced expression of Albatross (Fas-binding factor-1), also known for its capacity to bind K8/18 IFs. In addition, the K8/K18 loss affected the distribution of K5/K14-positive mTECs, but not their differentiation status. Together, the results indicate that K8/K18 IFs constitute key promoters of the thymic epithelium integrity.

## Introduction

Keratins (Ks), the intermediate filament (IF) proteins of epithelial cells, constitute a multigene family of acidic (or type I, K9 to K23) and basic proteins (or type II, K1 to K8 and K71 to K80), expressed as distinct pairs in a cell differentiation-dependent manner [1], [2]. For instance, the K5/K14 pair is present in the keratinocytes of the basal layer of stratified epithelia, whereas the terminally differentiated keratinocytes contain the K1/K10 pair. K8 and its partner K18 are the first IF proteins expressed in the embryo [3]–[6], where they become prominent in simple epithelium and periderm at the late stage, and then restricted to simple single-layered epithelia, such as lung and liver, at the post-natal stage [7].

Much of our knowledge on the multiple functions of K8/K18 IFs has derived from studies performed on K8-null mice generated via a targeted mutation in the germ line [8], [9]. Actually, a large part of the work has used hepatocytes as cell model, given that their IFs consist solely of the K8/K18 pair and that a loss of one keratin partner normally leads to the degradation of the other [10]. For instance, using cultured K8-knockout (K8-null) mouse hepatocytes and their wild-type (WT) counterparts, we have demonstrated that K8/K18 IFs contribute to the maintenance of the integrity of the hepatocyte surface membrane in response to mechanical stress [11], and modulate the hepatocyte response to various apoptotic stimuli [12]–[14]. Moreover, other lines of work on different K8-null versus WT mouse simple epithelial cells, such as those from intestine and pancreas, have revealed a K8/K18 IF involvement in epithelial cell polarity and protein targeting to subcellular compartments [15]–[16], in spite of the fact that such cell types contain the K8/K18 pair in combination with 1–2 other keratins. In this context, it appears that the K8/K18 pair may exert multiple functions within the same epithelial cell type.

Thymic epithelial cells (TECs) constitute a major component of thymic stroma, which provides a specialized microenvironment for survival, proliferation and differentiation of thymocytes [17] **.** Notably, TECs comprise two main types, referred to as cortical TECs (cTECs) and medullary TECs (mTECs), according to their regional confinement within the organ. Previous studies using Ks as markers, have revealed a K8 expression largely restricted to cTECs, with a small subset within the medulla versus a K5 expression mainly confined to the medulla, with a subset of K5+K8+ cells at the corticomedullary boundary and a scattered distribution in the cortex. Such differential keratin expression patterns have previously led to the proposal that K5+K8+ TECs constitute a population of immediate precursors to K5–K8+ cTECs [18], in line with recent studies indicating that both types of TECs arise through differentiation of a common progenitor [19], [20]; however, the keratin contribution to such a TEC differentiation is unknown.

In work reported here, we used K8-null and WT mice to address the involvement of K8/K18 IFs in the cellular organization and integrity of thymic epithelium, in terms of cTEC and mTEC structure and organization, and mTEC differentiation status. The results show that K8/K18 IFs are required for the prevention of apoptosis in cTECs and the maintenance of structural integrity in both TEC types.

## Materials and Methods

### Mice

Details on the establishment of the K8-deficient FVB/N mouse line have been reported previously [9]. K8-null mice and their WT littermates, in an FVB/N background, were housed in the specific pathogen free animal facility at our research center. Age- and sex-matched mice were used throughout the work. The Animal Care and Use Committee of the Centre de Recherche du CHU de Québec approved the animal experiments. Experiments were performed according to the rules of our institutional animal care and use committee.

### Thymus Weight

Mice were sacrificed via cervical dislocation, and thymi were dissected and then weighed.

### Immunofluorescence Histology

Thymus tissues were isolated and immersed in OCT compound. Frozen thymic sections were prepared and immunofluorescence staining was performed on thymic sections previously described [21]–[23]. The following antibodies and reagents were used: rabbit anti-K5 (Covance Research Products Inc.), mouse anti-K8 (Progen, Heidelberg, Germany), mouse anti-K18 (LE61; a gift of Dr. B. Lane at University of Dundee), rabbit anti-K14 (Covance Research Products Inc.), rat anti-E-cadherin (clone ECCD2) (Takara Bio Inc., Shiga, Japan), rat anti-Ly51 (Biolegend, San Diego, CA), rat anti-CD205 (Serotec, Kidlington, UK), rabbit anti-β5t [24], rabbit anti-Albatross [25], mouse anti-desmoplakins I & II (Progen), rabbit anti-Fas (Assay Design Inc., Ann Arbor, MI), rabbit anti-p63 (Santa Cruz Biotechnology), rat anti-CD80 (eBioscience), Rat anti-Aire mAb (RF33-1; a gift of Dr. M. Matsumoto at Tokushima University) and Alexa Fluor-labeled donkey secondary antibodies (Molecular Probes, Eugene, OR). The binding to biotinylated *Peanut Agglutinin* (PNA; Vector Laboratories, Burlingame, CA), biotinylated *Ulex europaeus agglutinin-1* (UEA-1; Vector Laboratories) or biotinylated *Tetragonolobus purpureas agglutinin* (TPA; Sigma-Aldrich) was followed by FITC-conjugated streptavidin (eBioscience). Confocal laser-scanning microscopy analysis was performed on a Zeiss LSM 510 (Carl Zeiss; Oberkochen, Germany). Negative controls were performed by replacement of first-step antibodies by isotype-matched monoclonal antibodies or species-matched antibodies. Representative images were chosen from each experiment for figure editing.

### Flow Cytometry

Fluorescence-conjugated reagents used for Flow cytometry: anti-CD3-FITC (555274), anti-CD4-PE-Cy7 (552775), abti-CD8a-APC-Cy7 (557654), anti-CD44-APC (559250), anti-CD25-PE (553075) and 7-amino-actinomycin D (7-AAD) staining solution were purchased from BD Biosciences (Mississauga, ON, Canada). Thymi were dissected and mechanically disrupted to release thymocytes as single cell suspensions. For each sample, 1×10^6^ cells were re-suspended in 100 µl of washing buffer (Phosphate buffered saline pH 7.4; bovine serum albumin 0.5%; EDTA 2 mM) and stained with 7-AAD and directly-labeled commercial antibodies at 4°C for 15 minutes. Unstained and full-minus-one (FMO) controls for each fluorophore were also performed. Compensation controls were performed using compensation beads (Rat anti mouse IgG-specific BD Compbead; BD Biosciences). Cells were washed three times with washing buffer and resuspended in 200 µl washing buffer, prior to acquisition on a FacsCantoA Flow cytometer driven by FacsDiva software (BD Biosciences, San 140 Jose, CA, USA). For each sample, 10^5^ live cells (7-AAD low/negative) were acquired. Data analysis was performed on FCS Express 3.0 software (Denovo Software, Los Angeles, CA).

### Apoptosis Assay

Apoptosis analysis was performed on thymic sections by TUNEL assay as previously described [26], [27] with slight modification. The positive signals were visualized by FITC-conjugated streptavidin. Apoptotic cells were also assessed for caspase-3 activation. After immunofluorescence staining for the activated form with anti-cleaved caspase-3 Ab (Trevigen, Gaithersburg, MD), active caspase-3-positive TECs were counted in six randomly chosen fields from each tissue section and plotted as the number of apoptotic cells/mm^3^ of cortical area.

### RNA Extraction and Semi-quantitative RT-PCR

Total RNA was extracted from thymus of individual mice using a SV Total RNA Isolation System kit (Promega Corporation, Madison, WI, USA) according to the manufacturer’s protocol. Semi-quantitative RT-PCR was carried out using a PrimeScript One-Step RT-PCR kit (Takara Bio Inc.) with the primers; Albatross sense primer, GACGAGACCCTCACCTTTGGGG; Albatross antisense primer, GCTCTGTTTGAAGCCTAGGTCT; β-actin sense primer, GTGGGCCGCTCTAGGCACCA; β-actin antisense primer, TGGCCTTAGGGTTCAGGGGGG. Amplification was performed with a denaturing temperature of 94°C, an annealing temperature of 60°C, and an extending temperature of 72°C, each for 1 min, followed by a final extension at 72°C for 8 min. The number of cycles was optimized for each product. The RT-PCR products were then analyzed using agarose gel electrophoresis and ethidium bromide staining.

### Statistics

Statistical analysis was performed with the nonparametric, unpaired Mann-Whitney test using Prism software. Probability values less than 0.05 were considered statistically significant.

## Results

### Altered Structure in TECs of K8-null Mice

**Figure 1:**
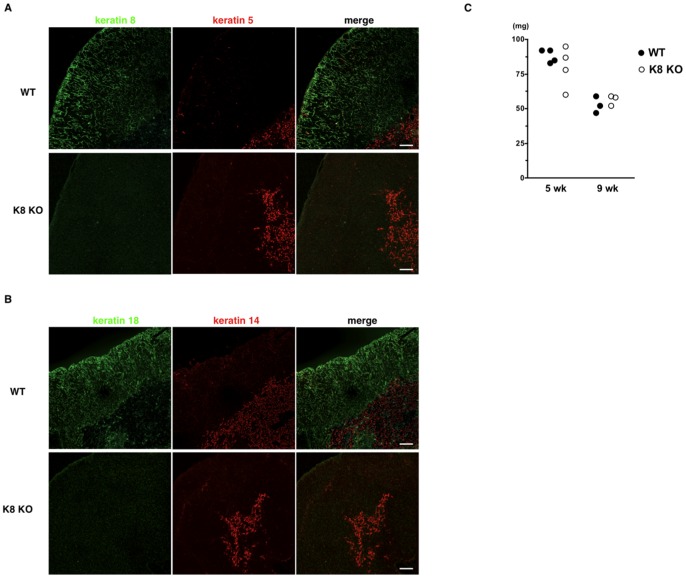
Keratin 8-deficient mice are absent from keratin 8/18 in thymic cortex. Immunofluorescence staining of thymus sections of 7-week-old wild-type FVB/N mice (WT) and K8-deficient FVB/N mice (K8 KO) was performed to detect K8 (**A**) or K18 (**B**) (green) and K5 (**A**) or K14 (**B**) (red). K8-deficient mice are lack of K8/K18 expression in the cortex. Note the altered morphology of K5+ or K14+ mTECs in K8-deficient mice. Data are representative of independent experiments (*n = *8 in each group). C, cortex; M, medulla. Scale bars = 100 µm. (**C**) Thymi from wild-type FVB/N mice and K8-deficient FVB/N mice at 5 weeks and 9 weeks of age were weighed. Each point provides the thymus weight of individual animal.

Keratin expression patterns have been used as indicators of TEC phenotype and differentiation status [18]. Here, we used WT and K8 null thymi to determine whether the loss of K8 may alter the differential expression of K5, K14 and K18 in cTECs and mTECs, as assessed by confocal imaging. We first confirmed that K8 in WT thymus was essentially found in all cTECs, and a small subset of cells in the medulla (Figure 1A), whereas K5 was prominent in mTECs; K5 expression was hardly detectable in the cortex. Moreover, as expected, the K18 detection pattern matched with that of K8 in the cortex, whereas K14 expression pattern compared well with that of K5 (Figure 1B). It is worth noticing that cTECs exhibited an irregular reticular organization. Notably, K8-null thymus displayed the normal cellular segregation in both the cortex and the medulla. Moreover, the K8 loss was confirmed (Figure 1A), along with the absence of K18 in the cortex (Figure 1B); a parallel analysis revealed that K5- and K14-expressing TECs remained restricted to the medulla. Of additional note, thymus weights of K8-null mice were comparable to those of WT mice (Figure 1C). Although the K8/K18 loss did not result to a thymic atrophy, it led to a reduction of the medullary areas containing K5+14+ mTECs, along with a concomitant increase in the K18-containing cortical areas (Figure 1A and B).

**Figure 2:**
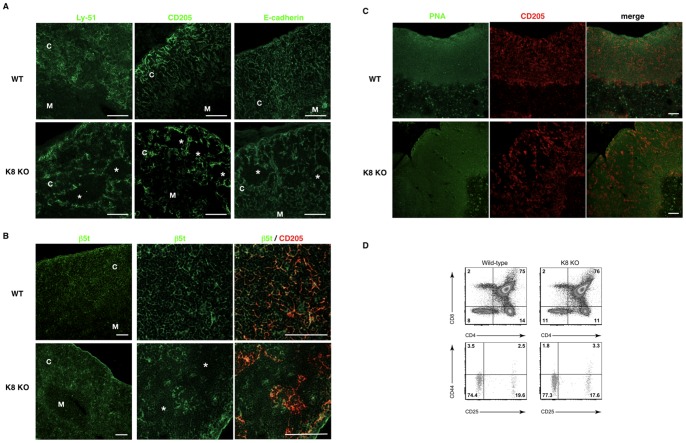
Altered morphology of cTECs in keratin 8-deficient mice. (**A**) Immunofluorescence staining of thymus sections of 7-week-old wild-type FVB/N mice (WT) and K8-deficient FVB/N mice (K8 KO) was performed to detect Ly-51, CD205 or E-cadherin (green). (**B**) Co-staining with β5t (green) and CD205 (red). The areas devoid of Ly-5, CD205, E-cadherin and β5t are seen in the cortex of K8-deficient mice. (**C**) Localization of immature thymocytes in K8-deficient thymus. Thymus sections of both strains were evaluated for binding of PNA (green). Note that immature thymocytes are localized in CD205+ cortex of both mice. Data are representative of independent experiments (*n = *8 in each group). C, cortex; M, medulla. Scale bars = 100 µm. (**D**) Flow cytometric analyses of thymocytes from 7-week-old wild-type FVB/N mice and K8-deficient FVB/N mice. Thymocytes were stained for CD4 and CD8 (top), and CD3, CD4 and CD8-triple-negative thymocytes in each mouse were stained for CD25 and CD44 (bottom). Numbers indicate percentages in each quadrant. Data are representative of independent experiments (*n = *4 in each group).

To further assess the impact of the K8/K18 loss on TEC heterogeneity, we compared the expression of non-keratin markers for cTECs in WT versus K8-null mice, namely Ly51 and CD205 [28], [29]. As shown in Figure 2A, both markers were restricted to the cortex in WT thymus. Moreover, as reported previously [30], the adherens junction protein E-cadherin was expressed in all TECs of both thymic tissue compartments (Figure 2A). In contrast, there were large areas devoid of DFC205, Ly-51 and E-cadherin stainings in the cortex of K8-null thymus, as result of the K8/K18 loss (Figure 2A). In the light of such findings, the cell type analysis was expanded to include a novel β subunit of the 20S proteasome, designated β5t, expressed in the majority of cTECs [24]. As shown in Figure 2B, β5t-expressing cells were present in the cortex of WT thymus, and the majority of these cells were also positive for CD205, confirming that β5t is primarily expressed in cTECs. In addition, the cortical areas devoid of CD205 were frequently β5t negative in K8-null thymus, as result of the K8/K18 loss. Still, a small number of β5t-positive cells were CD205 negative, suggesting that cells other than cTECs also express β5t.

Additionally, given that immature thymocytes, as identified with the expression of the lectin PNA ligand, are preferentially localized to the thymic cortex [31], we assessed their regional distribution in WT and K8-null thymi. As shown in Figure 2C, PNA-positive thymocytes distributed well in the cortex compartments from both WT and K8-null thymi, indicating that the K8/K18 loss did not affect the localization of immature thymocytes. In parallel, flow cytometric analyses performed on thymocytes revealed the appropriate relative percentages of double positive CD4^+^/CD8^+^ immature thymocytes and single CD8^+^ thymocytes in both WT and K8-null thymi (Figure 2D). In the same way, the relative expressions of CD25 and CD44 in CD3, CD4 and CD8-triple-negative thymocytes from K8-null mice were comparable to those from age-matched WT mice. Thus, thymocyte development appeared phenotypically normal in K8-null mice.

### Enhanced Apoptosis in cTECs of K8-null Mice

**Figure 3:**
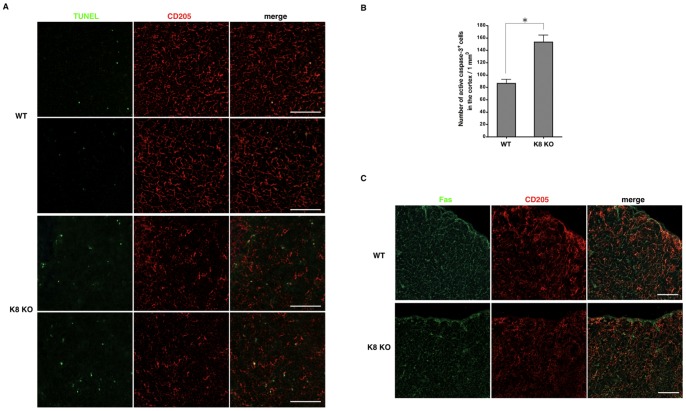
Apoptosis and Fas expression in the cTECs of keratin 8-deficient mice. Thymus sections of 7-week-old wild-type FVB/N mice (WT) and K8-deficient FVB/N mice (K8 KO) were evaluated for CD205 expression (**A**) and TUNEL assay (**A**), active caspase-3 (**B**) or Fas (**C**). (**A**) Arrows indicate apoptotic epithelial cells. (**A, C**) Data are representative of independent experiments (*n = *8 in each group). C, cortex; M, medulla. Scale bars = 100 µm. (**B**) Proportion of active caspase-3-positive cells was assessed as described in *Materials and Methods*. Data are shown as the mean ± SD (*n = *4 in each group). *p<0.05.

Previous studies have revealed that K8-null mice are highly sensitive to various apoptotic stimuli including Fas ligand [12], [13]. One possible explanation for the marked epithelial voids in K8-null thymus was a depletion of cTECs via apoptosis, and accordingly we looked for the presence of apoptotic cells in WT versus K8-null thymi, using the TUNEL assay. As shown in Figure 3A, apoptotic cells were detected along the boundary of the CD205-positive epithelial voids in K8-null thymus; apoptosis was hardly detected in the medullary region (data not shown). In addition, by staining for activated caspase-3, we found increased apoptotic cells in the cortex of K8-null thymi, indicating that apoptosis of the cortical thymic epithelium was increased in the absence of K8 (Figure 3B). We did not find any alternation of Fas receptor expression on cTECs of K8-null thymus (Figure 3C).

### Down Regulation of Albatross in cTEC of K8-null Mice

**Figure 4:**
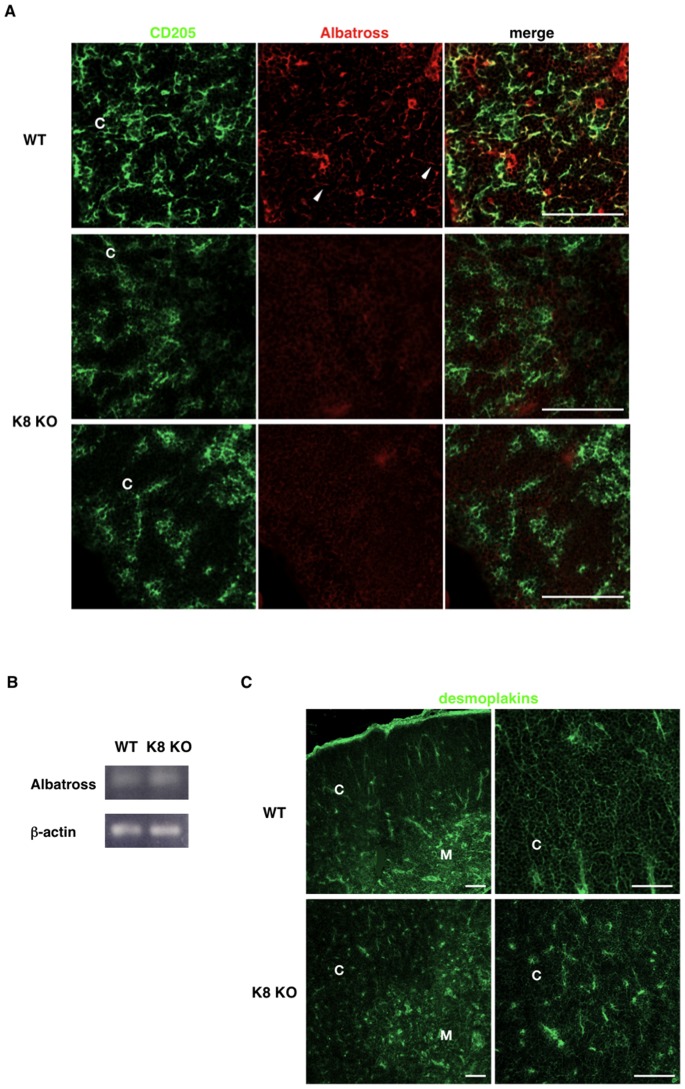
Expression of Albatross and desmoplakins in the thymi of keratin 8-deficient mice. Immunofluorescence staining of thymus sections of 7-week-old wild-type FVB/N mice (WT) and K8-deficient FVB/N mice (K8 KO) was performed to detect CD205 (**A**) or desmoplakins I/II (**C**) (green) and Albatross (**A**) (red). (**A**) Whereas colocalization of CD205 and Albatross is typically seen in wild-type, Albatross expression was reduced in the cortex of K8-deficient thymus. (**B**) Semi-quantitative RT-PCR analysis for Albatross was performed on Total RNA obtained from each thymus: *lower panel*, equal loading as defined by the levels of β-actin mRNA. (**C**) Localization of desmoplakins I/II is unaltered in K8 deficient thymus. Data are representative of independent experiments (*n = *8 in each group). C, cortex; M, medulla. Scale bars = 100 µm.

Albatross, also known as Fas-binding factor-1, was initially found to interact with Fas receptor [32]. We have previously shown that Albatross binds to K8/18 [25]. We thus compared the Albatross expression in cTECs of WT versus K8-null thymi. As shown in Figure 4A, Albatross co-localized with CD205-positive cTECs in WT-thymus, and its presence was reduced in cTECs of K8-null thymus. Additional analysis on Albatross expression using semi-quantitative RT-PCR revealed comparable mRNA levels in WT and K8-null thymi (Figure 4B). Together, these results indicate that the Albatross disappearance was due to a mis-regulation occurring at the post-translational level in K8/K18-lacking cTECs.

Desmosomes, as specialized cadherin-mediated adhesion complexes that impart stability and rigidity to tissues under mechanical stress [33], have been involved in epithelial cell morphogenesis and positioning [34], [35]. Moreover, since desmosomes link keratin IFs of one cell to those of its neighbours, together keratin IFs and desmosomes form an integrated and mechanically resilient network across the epithelium [36] **.** Furthermore, as cytolinkers, desmoplakins are responsible for anchoring the keratin IFs to the desmosome, and our previous work has revealed that K8/K18 regulate the desmoplakin deposition at desmosomes in hepatocytes [37]. We thus assessed the contribution of K8/K18 IFs to desmoplakin deposition in cTECs and mTECs. As shown in Figure 4C, desmoplakin-expressing cells were scatted in all regions of both WT and K8-null thymi, indicating that the K8/K18 loss did not affect the desmosome-mediated stability in TECs.

### mTEC Morphology and Differentiation in K8/K18-lacking Mice

**Figure 5:**
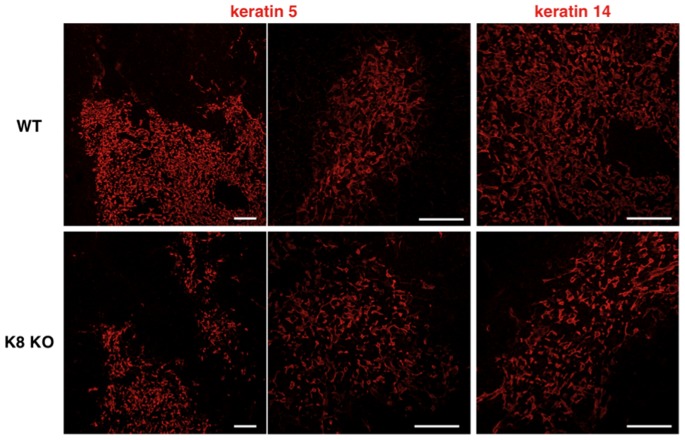
Altered morphology of mTECs in keratin 8-deficient mice. Immunofluorescence staining of thymus sections of 7-week-old wild-type FVB/N mice (WT) and K8-deficient FVB/N mice (K8 KO) was performed to detect K5 or K14 (red). K8-null thymus displays a marked reduction in the frequency of K5+ or K14+ cells. Higher magnification of K8 null thymus to show a looser distribution of K5+ or K14+ cells. Data are representative of independent experiments (*n = *8 in each group). C, cortex; M, medulla. Scale bars = 100 µm.

**Figure 6:**
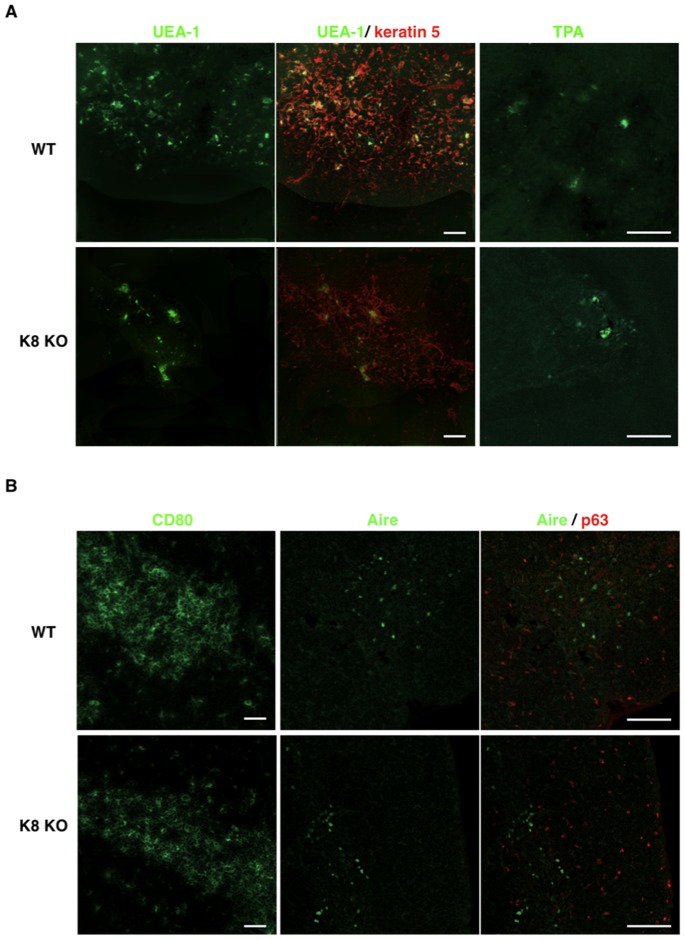
Development of mTECs in keratin 8-deficient mice. (**A**) Immunofluorescence staining of thymus sections of 7-week-old wild-type FVB/N mice and K8-deficient FVB/N mice was performed to detect the binding to either UEA-1 or TPA (green) and K5 (red). (**B**) Thymus sections of both strains were stained for the detection of either CD80 or Aire (green) with p63 (red). Data are representative of independent experiments (*n = *8 in each group). Scale bars = 100 µm.

Since K8-null thymus exhibited a smaller medulla (see Figure 1), we asked whether a K8/K18 loss could affect mTEC morphology. Intriguingly, K8-null thymus displayed a marked reduction in the frequency of K5- or K14-positive mTECs (Figure 5), in association with the formation of weak cell-cell contacts. In parallel, we assessed the differentiation status of mTECs in WT versus K8-null thymi, using UEA-1, TPA, CD80, Aire and p63 as relevant cell markers. The development of mTECs is accompanied by an increase in the expression levels of a carbohydrate epitope that binds the lectin UEA-1 [38]. As shown in Figure 6A, UEA-1 reacted with a subset of mTECs in both strains. It has been demonstrated that the fucose-specific lectin TPA particularly binds to Hassall’s corpuscles [38]. The binding of TPA was also restricted to mTECs, and the fractions of TPA-binding TECs were occasionally detected in small globular cell bodies (Figure 6A). Hassall’s corpuscles were also seen in the medulla of K8 null thymus. CD80, a marker for mature mTECs [39], was also distributed in the medulla of K8 null thymus (Figure 6B). A small population of mTECs expresses the autoimmune regulator Aire, which is crucial in the induction of T cell tolerance toward tissue-restricted antigen [40]. p63 is strongly expressed in epithelial stem cells of the thymus and specifically functions to maintain their extraordinary proliferative capacity [41]. Matched distributions in the medulla of WT versus K8-null thymus were also observed for Aire and pan-p63 (Figure 6B). All p63 isoforms are expressed in the normal thymus [42]. No alteration was detected in K8-null thymus for expression of all transactivation-active p63 (TAp63) and amino-deleted p63 (ΔNp63) isoforms in K8-null thymus, as assessed by RT-PCR (data not shown). On the whole, these results indicate that the K8/K18 loss led to alterations in mTEC morphology, without affecting their differentiation status.

## Discussion

The results reported here reveal that the loss of K8/K18 IFs altered the TEC structure and organization, namely a prominent cTEC depletion associated with an increased apoptosis, along with a perturbed mTEC distribution without a change in their differentiation status, demonstrating a K8/K18 IF involvement in the maintenance of thymic epithelium integrity. Still, the K8-null thymus remains divided into a peripheral cortex and a central medulla, thus indicating that K8 expression is dispensable for cell segregation between cortex and medulla. Moreover, the K8/K18 loss did not affect the localization of immature thymocytes in the cortex. Still, in line with previous work demonstrating that the loss of one keratin normally leads to the degradation of its partner [1], [2], [9], our data show that the K8 loss leads to the breakdown of K18 in cTECs.

The present findings demonstrate large areas devoid of the cTEC markers, CD205, Ly51 and β5t, within the cortex of K8-deficient thymus. Since most K8-positive cTECs also express CD205 and Ly51 (data not shown), it is unlikely that the subset of CD205/Ly51-negative cTECs undergoes an expansion in response to the K8/K18 loss. While we have reported that an histological evaluation of apoptotic cells *in vivo* is occasionally limited because the dying cells are rapidly engulfed and their DNA is degraded by macrophages [26], [27], the present results show enhanced TUNEL-positive signals in K8-null mice, particularly within the cTEC compartment. Actually, the epithelial voids in the cortex of K8-null thymus support previous reports demonstrating that keratins (including K8/K18) exert non-mechanical functions, including a protection from apoptosis [12], [13].

Keratin IFs are connected to cell-cell and cell-matrix junctions via cytoskeletal linker proteins [36] **.** The present data suggest that the K8/K18 loss leads to decreased Albatross protein expression in the thymus via a post-translational mechanism, in line with previous work with K8-transfectant cells showing an increased Albatross protein content that correlates with the increase in keratin expression [25], thus indicating that K8, likely in association with partner K18, regulates Albatross protein stability. However, while there is good evidence for an interaction of Albatross with the cytosolic domain of Fas receptor [32] and the involvement K8/K18 IFs as modulators of Fas density at cell surface [13], the present results indicate that the K8/K18 loss has little effect on Fas receptor expression in cTECs. While the function of Albatross for Fas receptor signaling is unknown, Albatross may suppress Fas receptor signaling. On this ground, the actual molecular mechanism underlying the interplay between K8/K18 IFs, Albatross and Fas receptor in these TECs remains unclear.

Intriguingly, the K8/K18 IF loss resulted in a reduced number of K5/K14-expressing mTECs, in association with alterations in cell shape and cell-cell contacts, but had no effect on the binding of lectins, UEA-1 and TPA, nor the expression of the mature mTEC markers, CD80 or Aire, thus affecting the morphology of K5/K14-expressing mTECs but not their differentiation status. Given that both types of TECs derive from a common progenitor [19], [20], it is likely that the progenitor cells are biased toward the fate of cTECs to compensate their depletion via apoptosis. A small subset of mTECs with a globular profile has been shown to express K8 but not K5 [18], but the present data using CD205 and Ly-51 to assess the fate of this mTEC subset in K8/K18-lacking thymus did not provide a definitive answer. Actually, part of the uncertainty comes from the fact that CD205+ or Ly-51+ cells are occasionally seen throughout the medulla of thymus, as these makers are also expressed in thymic dendritic cells. K8-null mice at 12 weeks of age following the onset of an inflammatory bowel disease [9], displayed the thymic architectural structures similar to those of 7-week old mice and did not exhibit early thymic atrophy (data not shown). Thus, the possibility that K8/K18 IFs are involved in molecular program(s) governing the differentiation of K5+K8+ TECs constitutes a challenging issue.

In conclusion, the present results uncover a key contribution of simple epithelium K8 to the maintenance of TEC structural integrity. While several genetic deficiencies are known to be associated with abnormal mTEC maturation, few reports have described abnormalities in cTEC development [43]. Here, we show that K8-deficient mice display aberrant structures in both cTECs and mTECs. TECs play a critical role in the generation of a functional and self-tolerant T-cell repertoire [44]. Considering that K8-deficient mice develop colorectal hyperplasia accompanied by a pronounced inflammation of the lamina propria and submucosa [9], a relevant functional perspective is to clarify the T cell repertoire and the associated immune responses in K8-null mice.

## References

[1] Coulombe PA, Omary MB (2002) ‘Hard’ and ‘soft’ principles defining the structure, function and regulation of keratin intermediate filaments. Curr Opin Cell Biol 14: 110–122. 11792552 10.1016/s0955-0674(01)00301-5

[2] Bragulla HH, Homberger DG (2009) Structure and functions of keratin proteins in simple, stratified, keratinized and cornified epithelia. J Anat 214: 516–559. 19422428 10.1111/j.1469-7580.2009.01066.x PMC2736122

[3] Jackson BW, Grund C, Schmid E, Bürki K, Franke WW, et al (1980) Formation of cytoskeletal elements during mouse embryogenesis. Intermediate filaments of the cytokeratin type and desmosomes in preimplantation embryos. Differentiation 17: 161–179. 6161051 10.1111/j.1432-0436.1980.tb01093.x

[4] Brulet P, Babinet C, Kemler R, Jacob F (1980) Monoclonal antibodies against trophectoderm-specific markers during mouse blastocyst formation. Proc Natl Acad Sci U S A 77: 4113–4117. 6933460 10.1073/pnas.77.7.4113 PMC349780

[5] Moll R, Franke WW, Schiller DL, Geiger B, Krepler R (1982) The catalog of human cytokeratins: patterns of expression in normal epithelia, tumors and cultured cells. Cell 31: 11–24. 6186379 10.1016/0092-8674(82)90400-7

[6] Lane EB, Goodman SL, Trejdosiewicz LK (1982) Disruption of the keratin filament network during epithelial cell division. EMBO J 1: 1365–1372. 6202508 10.1002/j.1460-2075.1982.tb01324.x PMC553218

[7] Oshima RG, Baribault H, Caulín C (1996) Oncogenic regulation and function of keratins 8 and 18. Cancer Metastasis Rev 15: 445–471. 9034603 10.1007/BF00054012

[8] Baribault H, Price J, Miyai K, Oshima RG (1992) Mid-gestational lethality in mice lacking keratin 8. Genes Dev 7: 1991–2002. 10.1101/gad.7.7a.1191 7686525

[9] Baribault H, Penner J, lozzo RV, Wilson-Heiner M (1994) Colorectal hyperplasia and inflammation in keratin 8-deficient FVB/N mice. Genes Dev 8: 2964–2973. 7528156 10.1101/gad.8.24.2964

[10] Marceau N, Gilbert S, Loranger A (2004) Uncovering the roles of intermediate filaments in apoptosis. Methods Cell Biol 78: 95–129. 15646617 10.1016/s0091-679x(04)78005-x

[11] Loranger A, Duclos S, Grenier A, Price J, Wilson-Heiner M, et al (1997) Simple epithelium keratins are required for maintenance of hepatocyte integrity. Am J Pathol 151: 1673–83. 9403718 PMC1858351

[12] Caulin CC, Ware F, Magin TM, Oshima RG (2000) Keratin-dependent, epithelial resistance to tumor necrosis factor-induced apoptosis. J Cell Biol 149: 17–22. 10747083 10.1083/jcb.149.1.17 PMC2175089

[13] Gilbert S, Loranger A, Daigle N, Marceau N (2001) Simple epithelium keratins 8 and 18 provide resistance to Fas-mediated apoptosis. The protection occurs through a receptor-targeting modulation. J Cell Biol 154: 763–773. 11514590 10.1083/jcb.200102130 PMC2196458

[14] Habtezion A, Toivola DM, Asghar MN, Kronmal GS, Brooks JD, et al (2011) Absence of keratin 8 confers a paradoxicamicroflora-dependent resistance to apoptosis in the colon. Proc Natl Acad Sci U S A 108: 1445–1450. 21220329 10.1073/pnas.1010833108 PMC3029736

[15] Oriolo AS, Wald FA, Ramsauer VP, Salas PJ (2007) Intermediate filaments: a role in epithelial polarity. Exp Cell Res 313: 2255–2264. 17425955 10.1016/j.yexcr.2007.02.030 PMC1986643

[16] Toivola DM, Tao GZ, Habtezion A, Liao J, Omary MB (2005) Beyond cellular integrity: organelle-related and protein-targeting functions of intermediate filaments. Trends Cell Biol 15: 608–617. 16202602 10.1016/j.tcb.2005.09.004

[17] Farr AG, Dooley UL, Erickson M (2002) Organization of thymic medullary epithelial heterogeneity: implications for mechanisms of epithelial differentiation. Immunol Rev 189: 20–27. 12445262 10.1034/j.1600-065x.2002.18903.x

[18] Klug DB, Carter C, Crouch E, Roop D, Conti CJ, et al (1998) Interdependence of cortical thymic epithelial cell differentiation and T-lineage commitment. Proc Natl Acad Sci U S A 95: 11822–11827. 9751749 10.1073/pnas.95.20.11822 PMC21724

[19] Bleul CC, Corbeaux T, Fisch AP, Mönting JS, Boehm T (2006) Formation of a functional thymus initiated by a postnatal epithelial progenitor cell. Nature 441: 992–996. 16791198 10.1038/nature04850

[20] Rossi SW, Jenkinson WE, Anderson G, Jenkinson EJ (2006) Clonal analysis reveals a common progenitor for thymic cortical and medullary epithelium. Nature 441: 988–991. 16791197 10.1038/nature04813

[21] Odaka C, Morisada T, Oike Y, Suda T (2006) Distribution of lymphatic vessels in mouse thymus: immunofluorescence analysis. Cell Tissue Res 325: 13–22. 16541287 10.1007/s00441-005-0139-3

[22] Odaka C (2009) Localization of mesenchymal cells in adult mouse thymus: their abnormal distribution in mice with disorganization of thymic medullary epithelium. J Histochem Cytochem 57: 373–382. 19110482 10.1369/jhc.2008.952895 PMC2664980

[23] Bravo-Nuevo A, O’Donnell R, Rosendahl A, Chung JH, Benjamin LE, et al (2011) RhoB deficiency in thymic medullary epithelium leads to early thymic atrophy. Int Immunol 23: 593–600. 21865151 10.1093/intimm/dxr064 PMC3182298

[24] Murata S, Sasaki K, Kishimoto T, Niwa S, Hayashi H, et al (2007) Regulation of CD8+ T cell development by thymus-specific proteasomes. Science 316: 1349–1353. 17540904 10.1126/science.1141915

[25] Sugimoto M, Inoko A, Shiromizu T, Nakayama M, Zou P, et al (2008) The keratin-binding protein Albatross regulates polarization of epithelial cells. J Cell Biol 183: 19–28. 18838552 10.1083/jcb.200803133 PMC2557036

[26] Odaka C, Mizuochi T (1999) Role of macrophage lysosomal enzymes in the degradation of nucleosomes of apoptotic cells. J Immunol 163: 5346–5352. 10553058

[27] Odaka C, Mizuochi T (2002) Macrophages are involved in DNA degradation of apoptotic cells in murine thymus after administration of hydrocortisone. Cell Death Differ 9: 104–112. 11840161 10.1038/sj.cdd.4400941

[28] Rouse RV, Bolin LM, Bender JR, Kyewski BA (1998) Monoclonal antibodies reactive with subsets of mouse and humans thymic epithelial cells. J Histochem Cytochem 36: 1511–1519. 10.1177/36.12.2461413 2461413

[29] Jiang W, Swiggard WJ, Heufler C, Peng M, Mirza A, et al (1995) Nussenzweig MC: The receptor DEC-205 expressed bydendritic cells and thymic epithelial cells is involved in antigen processing. Nature 375: 151–155. 7753172 10.1038/375151a0

[30] Lee MG, Sharrow SO, Farr AG, Singer A, Udey MC (1994) Expression of the homotypic adhesion molecule E-cadherin by immature murine thymocytes and thymic epithelial cells. J Immunol 152: 5653–5659. 8207198

[31] Reisner Y, Linker-Israeli M, Sharon N (1976) Separation of mouse thymocytes into two subpopulations by the use of peanut agglutinin. Cell Immunol 25: 129–134. 9202 10.1016/0008-8749(76)90103-9

[32] Schmidt T, Karsunky H, Frass B, Baum W, Denzel A, et al (2000) A novel protein (Fbf-1) that binds to CD95/APO-1/FAS and shows sequence similarity to trichohyalin and plectin. Biochim Biophys Acta 1493: 249–254. 10978533 10.1016/s0167-4781(00)00163-9

[33] Garrod DR, Tselepis C, Runswick SK, North AJ, Wallis SR, et al (1999) Desmosomal adhesion. Adv Mol Cell Biol 28: 165–202.

[34] Runswick SK, O’Hare MJ, Jones L, Streuli CH, Garrod DR (2001) Desmosomal adhesion regulates epithelial morphogenesis and cell positioning. Nat Cell Biol 3: 823–830. 11533662 10.1038/ncb0901-823

[35] Vasioukhin V, Bowers E, Bauer C, Degenstein L, Fuchs E (2001) Desmoplakin is essential in epidermal sheet formation. Nat Cell Biol 3: 1076–1085. 11781569 10.1038/ncb1201-1076

[36] Godsel LM, Hsieh SN, Amargo EV, Bass AE, Pascoe-McGillicuddy LT, et al (2005) Desmoplakin assembly dynamics in four dimensions: multiple phases differentially regulated by intermediate filaments and actin. J Cell Biol 171: 1045–59. 16365169 10.1083/jcb.200510038 PMC2171300

[37] Loranger A, Gilbert S, Brouard JS, Magin TM, Marceau N (2006) Keratin 8 modulation of desmoplakin deposition at desmosomes in hepatocytes. Exp Cell Res 312: 4108–4119. 17126832 10.1016/j.yexcr.2006.09.031

[38] Farr AG, Anderson SK (1985) Epithelial heterogeneity in the murine thymus: fucose-specific lectins bind medullary epithelial cells. J Immunol 134: 2971–2977. 3856612

[39] Degermann S, Surh CD, Glimcher LH, Sprent J, Lo D (1994) B7 expression on thymic medullary epithelium correlates with epithelium-mediated deletion of V beta 5+ thymocytes. J Immunol 152: 3254–3263. 7511640

[40] Anderson MS, Venanzi ES, Klein L, Chen Z, Berzins SP, et al (2002) Projection of an immunological self shadow within the thymus by the aire protein. Science 298: 1395–1401. 12376594 10.1126/science.1075958

[41] Senoo M, Pinto F, Crum CP, McKeon F (2007) P63 is essential for the proliferative potential of stem cells in stratified epithelia. Cell 129: 523–536. 17482546 10.1016/j.cell.2007.02.045

[42] Candi E, Rufini A, Terrinoni A, Giamboi-Miraglia A, Lena AM, et al (2007) ΔNp63 regulates thymic development through enhanced expression of FgfR2 and Jag2. Proc Natl Acad Sci U. S. A. 104: 11999–2004. 10.1073/pnas.0703458104 PMC1924561 17626181

[43] Irla M, Hollander G, Reith W (2010) Control of central self-tolerance induction by autoreactive CD4+ thymocytes. Trends Immunol 31: 71–79. 20004147 10.1016/j.it.2009.11.002

[44] Starr TK, Jameson SC, Hogquist KA (2003) Positive and negative selection of T cells. Annu Rev Immunol 21: 139–176. 12414722 10.1146/annurev.immunol.21.120601.141107

